# (2*R*)-4-[(9*H*-Fluoren-9-ylmeth­oxy)carbon­yl]-2-methyl­piperazin-1-ium chloride

**DOI:** 10.1107/S1600536811048306

**Published:** 2011-11-19

**Authors:** Anne Ertan, Parhalad Sharma, Dokka Nagaraju, K. Deepthi, Sulur G. Manjunatha

**Affiliations:** a Pharmaceutical Development, AstraZeneca R&D, S-151 85 Södertälje, Sweden; b Pharmaceutical Development, AstraZeneca R&D, Off Bellary Road, Hebbal, Bangalore 560 024, India

## Abstract

The synthesis of the title salt, C_20_H_23_N_2_O_2_^+^·Cl^−^, was carried out with a precursor of known absolute configuration (*R*) and the X-ray analysis confirmed that the product retained the absolute configuration. In the crystal, the dominant packing motif is a chain running along [010] generated by N—H⋯Cl hydrogen bonding. C—H⋯O and C—H⋯Cl inter­actions are also observed.

## Related literature

For the use of piperazine and substituted piperazines as good linkers to pharmacophores in attempts to obtain compounds with desired pharmacokinetic and pharmacological properties, see: Cho *et al.* (2010 ▶); Wang *et al.* (2009 ▶). For packing coefficients, see: Kitaigorodskij (1973 ▶).
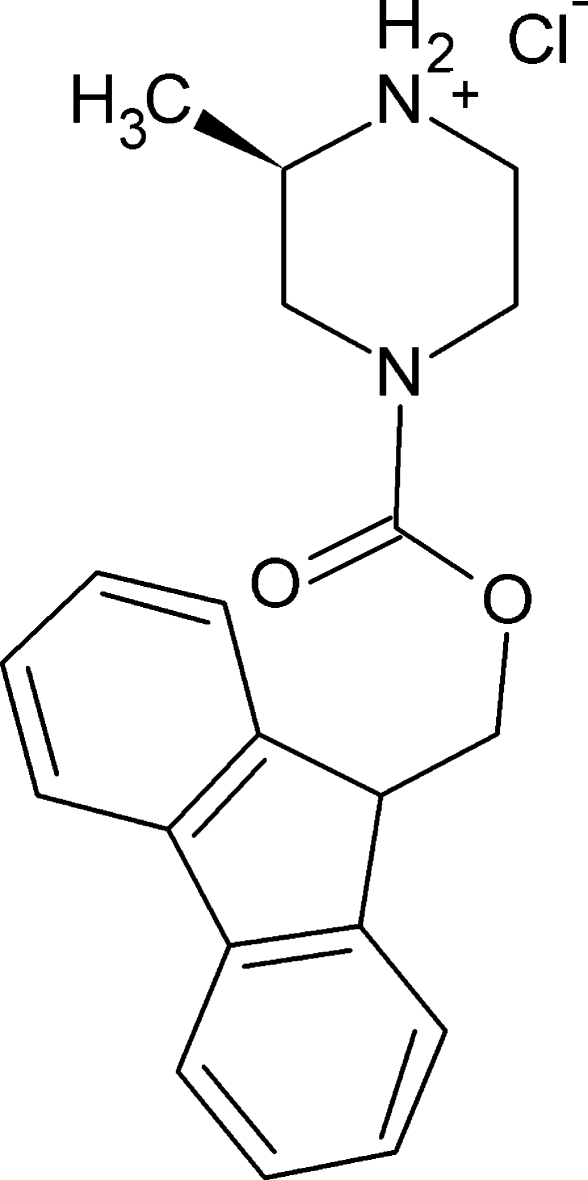


## Experimental

### 

#### Crystal data

C_20_H_23_N_2_O_2_^+^·Cl^−^
*M* *_r_* = 358.85
Monoclinic, 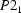

*a* = 8.3492 (3) Å
*b* = 7.4954 (2) Å
*c* = 14.9246 (3) Å
β = 90.6740 (18)°
*V* = 933.93 (5) Å^3^
*Z* = 2
Mo *K*α radiation
μ = 0.22 mm^−1^
*T* = 293 K
0.28 × 0.20 × 0.08 mm

#### Data collection

Nonius KappaCCD diffractometer
4227 measured reflections
4221 independent reflections
3684 reflections with *I* > 2σ(*I*)
*R*_int_ = 0.022

#### Refinement

*R*[*F* ^2^ > 2σ(*F* ^2^)] = 0.036
*wR*(*F* ^2^) = 0.086
*S* = 1.03
4221 reflections
227 parameters
1 restraint
H-atom parameters constrained
Δρ_max_ = 0.16 e Å^−3^
Δρ_min_ = −0.15 e Å^−3^
Absolute structure: Flack (1983 ▶), 1922 Friedel pairs
Flack parameter: −0.04 (5)

### 

Data collection: *COLLECT* (Nonius, 1998 ▶); cell refinement: *SCALEPACK* (Otwinowski & Minor, 1997 ▶); data reduction: *DENZO* (Otwinowski & Minor, 1997 ▶) and *SCALEPACK*; program(s) used to solve structure: *SIR97* (Altomare *et al.*, 1999 ▶); program(s) used to refine structure: initial refinement: *maXus* (MacKay *et al.*, 2000 ▶); final refinement: *SHELXL97* (Sheldrick, 2008 ▶); molecular graphics: *PLATON* (Spek, 2009 ▶) and *ORTEPII* (Johnson, 1976 ▶); software used to prepare material for publication: *PLATON* and *ACD/Labs* (ACD, 2011 ▶).

## Supplementary Material

Crystal structure: contains datablock(s) global, I. DOI: 10.1107/S1600536811048306/kp2361sup1.cif

Structure factors: contains datablock(s) I. DOI: 10.1107/S1600536811048306/kp2361Isup2.hkl

Supplementary material file. DOI: 10.1107/S1600536811048306/kp2361Isup3.cml

Additional supplementary materials:  crystallographic information; 3D view; checkCIF report

## Figures and Tables

**Table 1:** Hydrogen-bond geometry (Å, °)

*D*—H⋯*A*	*D*—H	H⋯*A*	*D*⋯*A*	*D*—H⋯*A*
N1—H1*A*⋯Cl1^i^	1.06	2.08	3.117 (2)	165
N1—H1*B*⋯Cl1^ii^	0.88	2.26	3.135 (2)	171
C2—H2*B*⋯O8^iii^	0.96	2.41	3.316 (2)	157
C21—H21⋯O8^iv^	0.98	2.54	3.469 (2)	158
C22—H22⋯Cl1	0.96	2.72	3.622 (2)	157

Symmetry codes: (i) 
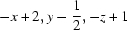
; (ii) 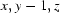; (iii) 
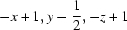
; (iv) 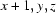.

**Fig. 1.**
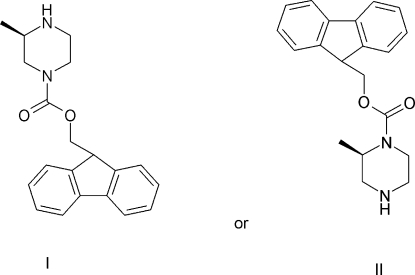
Scheme I and II showing the two possible mono protected products, not possible to elucidate their structures by chromatographic or spectroscopic analysis.

**Fig. 2.**
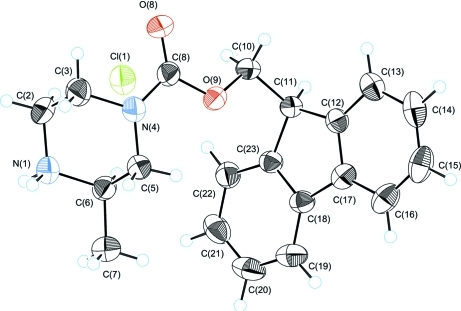
View of the title compound showing the atom-labelling scheme. Displacement ellipsoids of non-H atoms are drawn at the 50% probability level. ORTEPII (Johnson, 1976).

**Fig. 3.**
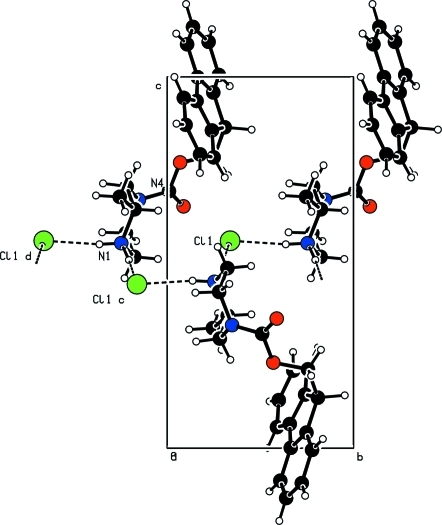
Part of the molecular H-bond scheme with the molecules joined as chains containg equivalent symmetry translated units along the [1 0 0] direction. Dotted lines indicate H-bond interactions. The view is along the [1 0 0] direction. PLATON (Spek, 2009).

**Fig. 4.**
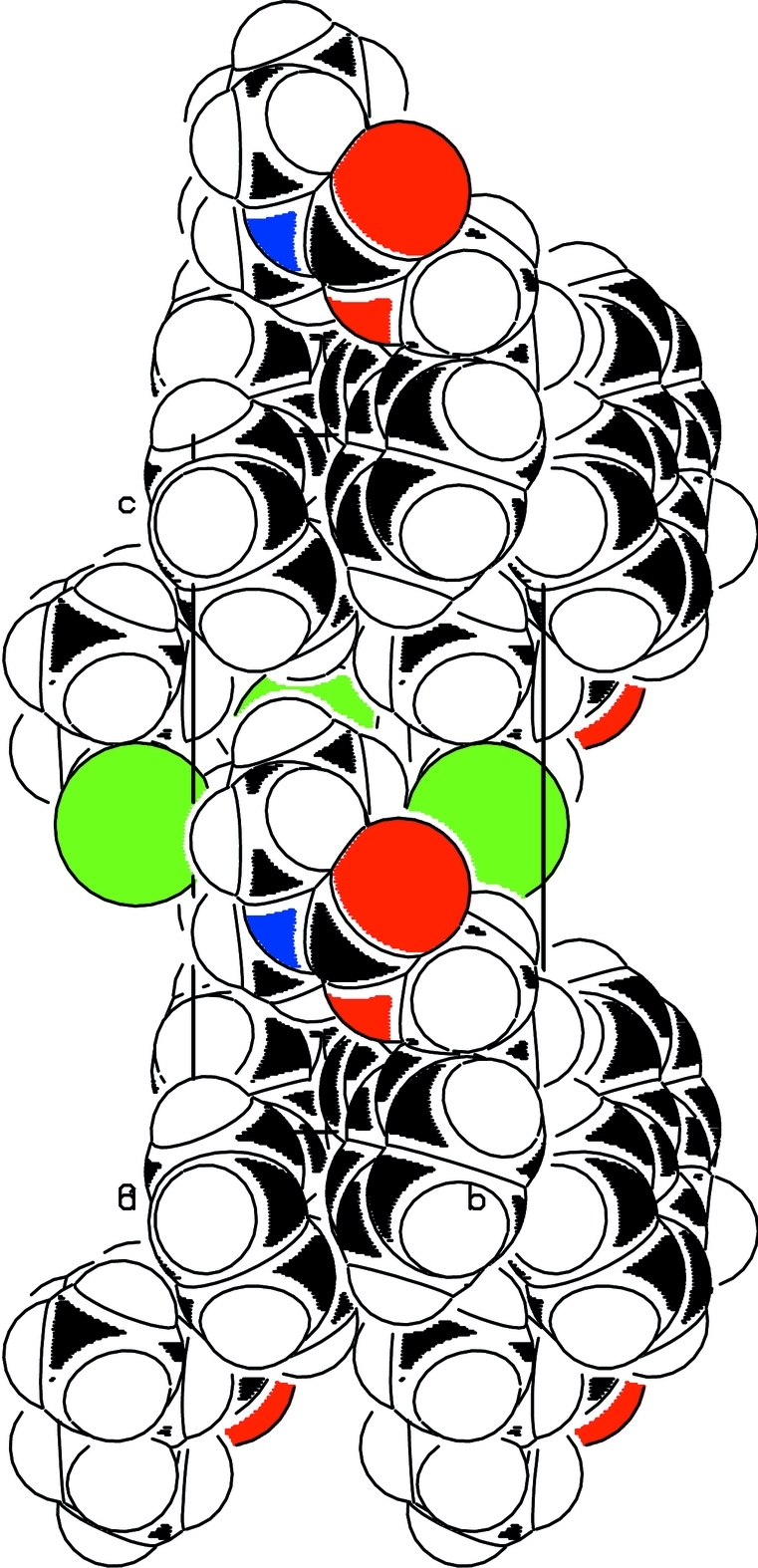
Spacefill packing diagram of the molecules in the unit cell along the [1 0 0] direction reflecting an efficient molecular packing arrangement. PLATON (Spek, 2009).

## supplementary crystallographic information

### Comment

Piperazine and substituted piperazines are good linkers to pharmacophores to bring out drug substance with desired pharmacokinetics and pharmacological properties (Cho *et al.*, 2010; Wang *et al.*, 2009). In this context there is a great need in selective mono N-protectected piperazines and efficient synthetic procedures for their preparation. As a part of our research in drug development we were in need of selective mono protection of 2-methylpiperazines. We could develop a simple procedure for the mono N-protection of 2-methylpiperazine with F-moc. The chromatographic and spectroscopic analysis indicated that the process is highly regioselective to give the mono protected product but the same techniques were inadequate to elucidate the structure (Fig. 1, Scheme I & II, ACD/Labs,1994–2011), therefore single-crystal technique was employed. The X-ray investigation of the title compound was undertaken to verify the structure and confirm the absolute stereochemistry of the intermediate made in the course of synthesis (Fig. 2). The absolute configuration around the chiral carbon atom C6 was determined to be 6*R*, which was expected from the synthesis using a precursor of *R*-configuration. The Flack's *x* parameter (Flack, 1983) was refined to -0.04 (5). The molecule has four hetero-atoms of which only one is protonated, the N1 atom. This potential H-bond donor participates in intermolecular H-bond interaction with the chloride ion (Table 1, Fig. 3), linking the molecules into infinite chain by N—H···Cl (chloride) interactions along the [0 1 0] direction (Fig. 3). Themolecules are efficiently packed, with no residual void for solvent inclusion (Fig. 4). The packing coefficient of I, calculated by *PLATON*, is 66.7% (Kitaigorodskij, 1973), reflecting an efficient molecular packing arrangement.

### Experimental

The chemicals used for the synthesis are purchased from: (*R*)-2-methyl piperazine from Manjing Gaungtong Pharmaceutical & Chemical Co. Ltd. and F-moc chloride from Spectrochem India Ltd.

Preparation of (2*R*)-4-[(9*H*-fluoren-9-ylmethoxy)carbonyl]-2- methylpiperazine-1-ium chloride

A solution of F-moc chloride (11.6 g, 0.0449 mol) in acetone (100 mL) was added drop wise to a solution of 2(*R*)-methyl piperazine (5.0 g, 0.0499 mol) in acetone (75 mL) at 2893 K over a period of 1.7 h. The temperature of the reaction mass was raised from 295 to 298 K and was allowed to stir for 1.5 h. The resulting solid was collected by filtration and washed with acetone to give 9.89 g (yield: 55.2% *w*/*w*) of the title compound as a white solid in form of hydrochloride salt. HPLC purity >98% and *M*+1: 323, melting point: 412 K (DSC thermogram).

Crystallization process

The single-crystal of (2*R*)-4-[(9*H*-fluoren-9-ylmethoxy)carbonyl]-2- methylpiperazin-1-ium chloride has been grown using vapour diffusion method: (2*R*)-4-[(9*H*-fluoren-9-ylmethoxy)carbonyl]-2-methylpiperazine-1-ium chloride (0.5 g) is dissolved in methanol (20 mL) in a small vial, which is placed inside a larger vial containing a small volume of a heptane (100 mL) in which the sample is insoluble. The larger vial is then sealed but the smaller one is open for the second solvent to intrude. The unit is kept as such for a period of 72 h. The obtained solid was collected by filtration and examined under microscope. A large block-shaped crystal of compound I, grown from methanol/heptane, was used for single-crystal X-ray diffraction experiment.

### Refinement

Data collection, structure solution and refinement:

Diffraction data for compound 1 was collected at RT using a Nonius Kappa-CCD diffractometer (Nonius, 1998), with graphite-monochromated Mo *K*α radiation (0.71073 Å). Details of X-ray experiment are summarized in Supplementary Material. The structure was solved by direct methods (Altomare *et al.*, 1999, SIR97) and refined with F^2^ against all reflections. The title compound 1 had one molecule in the asymmetric unit. The absolute configuration at the chiral C6 carbon atom was determined to  be R. The number of Friedel pairs measured was 1922. All non-H atoms were anisotropically refined. Although identified in late difference Fourier maps, the aromatic- and methyl-H atoms were calculated due to poor bond angles and constrained to ideal geometry positions with distance 0.96-0.98 Å, from the parent atoms. The H1A and H1B atoms, found from difference Fourier map, were refined a few cycles with isotropic displacement parameters but were constrained in the final cycles of refinement to 1.06 and 0.86 Å from their parent atoms. Due to the fact that both these H-atoms are involved in H-bonds, the refined positions were kept in the final structure model. The H atoms were refined using a riding model, with *U*_iso_(H) = 1.2*U*_eq_(C). Six low integer reflections shadowed by the beam stop were omitted from the final calculations. The highest residual electron density peak was located close to carbon C11 atom and the deepest hole close to carbon C10 atom. The original structure model was obtained (Altomare, SIR97) and refined initially within *maXus* software suite (MacKay *et al.*, 2000) but the final refinement of the structure was done with *SHELXL97* (Sheldrick, 2008).

### Crystal data

**Table:**

C_20_H_23_N_2_O_2_^+^·Cl^−^	*F*(000) = 380
*M**_r_* = 358.85	*D*_x_ = 1.276 Mg m^−^^3^
Monoclinic, *P*2_1_	Mo *K*α radiation, λ = 0.71073 Å
Hall symbol: P 2yb	Cell parameters from 2263 reflections
*a* = 8.3492 (3) Å	θ = 1.0–27.5°
*b* = 7.4954 (2) Å	µ = 0.22 mm^−^^1^
*c* = 14.9246 (3) Å	*T* = 293 K
β = 90.6740 (18)°	Block, colourless
*V* = 933.93 (5) Å^3^	0.28 × 0.20 × 0.08 mm
*Z* = 2	

### Data collection

**Table:**

Nonius KappaCCD diffractometer	3684 reflections with *I* > 2σ(*I*)
Radiation source: fine-focus	*R*_int_ = 0.022
graphite	θ_max_ = 27.5°, θ_min_ = 3.0°
φ and ω scans with κ offsets	*h* = −10→10
4227 measured reflections	*k* = −9→9
4221 independent reflections	*l* = −19→19

### Refinement

**Table:**

Refinement on *F*^2^	Hydrogen site location: inferred from neighbouring sites
Least-squares matrix: full	H-atom parameters constrained
*R*[*F*^2^ > 2σ(*F*^2^)] = 0.036	*w* = 1/[σ^2^(*F*_o_^2^) + (0.037*P*)^2^ + 0.176*P*] where *P* = (*F*_o_^2^ + 2*F*_c_^2^)/3
*wR*(*F*^2^) = 0.086	(Δ/σ)_max_ < 0.001
*S* = 1.03	Δρ_max_ = 0.16 e Å^−^^3^
4221 reflections	Δρ_min_ = −0.15 e Å^−^^3^
227 parameters	Extinction correction: *SHELXL97* (Sheldrick, 2008), FC^*^=KFC[1+0.001XFC^2^Λ^3^/SIN(2Θ)]^-1/4^
1 restraint	Extinction coefficient: 0.075 (4)
Primary atom site location: structure-invariant direct methods	Absolute structure: Flack (1983), 1922 Friedel pairs
Secondary atom site location: difference Fourier map	Flack parameter: −0.04 (5)

### Special details

**Table:**

Experimental. Crystals were crystallized from methanol/heptane by vapour diffusion.Number of collected frames: 212 Number of repeats: 1 Crystal-Detector distance (mm): 30 Exposure time (sec) per frame: 5 Phi-rotation (°) step: 2
Geometry. Bond distances, angles *etc*. have been calculated using the rounded fractional coordinates. All su's are estimated from the variances of the (full) variance-covariance matrix. The cell e.s.d.'s are taken into account in the estimation of distances, angles and torsion angles
Refinement. Refinement on *F*^2^ for ALL reflections except those flagged by the user for potential systematic errors. Weighted *R*-factors *wR* and all goodnesses of fit *S* are based on *F*^2^, conventional *R*-factors *R* are based on *F*, with *F* set to zero for negative *F*^2^. The observed criterion of *F*^2^ > σ(*F*^2^) is used only for calculating -*R*-factor-obs *etc*. and is not relevant to the choice of reflections for refinement. *R*-factors based on *F*^2^ are statistically about twice as large as those based on *F*, and *R*-factors based on ALL data will be even larger.

### Fractional atomic coordinates and isotropic or equivalent isotropic displacement parameters (Å^2^)

**Table:**

	*x*	*y*	*z*	*U*_iso_*/*U*_eq_	
O8	0.44893 (17)	0.0878 (2)	0.65110 (9)	0.0556 (5)	
O9	0.61979 (15)	0.06962 (17)	0.76938 (8)	0.0404 (4)	
N1	0.86676 (19)	−0.2463 (2)	0.55195 (9)	0.0431 (5)	
N4	0.61615 (18)	−0.1517 (3)	0.66869 (10)	0.0468 (5)	
C2	0.7114 (2)	−0.1768 (3)	0.51533 (12)	0.0512 (6)	
C3	0.5787 (2)	−0.2213 (3)	0.57921 (13)	0.0540 (7)	
C5	0.7678 (2)	−0.2172 (3)	0.70499 (11)	0.0438 (6)	
C6	0.9062 (2)	−0.1750 (3)	0.64313 (11)	0.0397 (5)	
C7	1.0634 (2)	−0.2547 (3)	0.67673 (14)	0.0532 (7)	
C8	0.5532 (2)	0.0098 (3)	0.69198 (11)	0.0398 (5)	
C10	0.5939 (2)	0.2555 (2)	0.78753 (11)	0.0380 (5)	
C11	0.70593 (19)	0.3087 (2)	0.86403 (10)	0.0336 (5)	
C12	0.6628 (2)	0.2475 (2)	0.95797 (11)	0.0358 (5)	
C13	0.5230 (2)	0.2774 (3)	1.00485 (13)	0.0446 (6)	
C14	0.5175 (3)	0.2224 (3)	1.09407 (13)	0.0529 (7)	
C15	0.6472 (3)	0.1385 (3)	1.13435 (13)	0.0571 (7)	
C16	0.7860 (3)	0.1070 (3)	1.08760 (13)	0.0514 (7)	
C17	0.7936 (2)	0.1632 (2)	0.99900 (11)	0.0389 (5)	
C18	0.9257 (2)	0.1579 (2)	0.93493 (11)	0.0370 (5)	
C19	1.0795 (2)	0.0892 (3)	0.94323 (15)	0.0504 (7)	
C20	1.1808 (2)	0.0990 (3)	0.87068 (16)	0.0555 (7)	
C21	1.1300 (2)	0.1765 (3)	0.79113 (15)	0.0528 (7)	
C22	0.9768 (2)	0.2495 (3)	0.78302 (12)	0.0429 (5)	
C23	0.87605 (19)	0.2402 (2)	0.85559 (11)	0.0349 (5)	
Cl1	0.82783 (6)	0.33785 (7)	0.55846 (3)	0.0512 (2)	
H1A	0.95740	−0.20530	0.50700	0.0520*	
H1B	0.86370	−0.36370	0.54900	0.0520*	
H2A	0.71940	−0.04950	0.50940	0.0620*	
H2B	0.68890	−0.22870	0.45770	0.0620*	
H3A	0.56490	−0.34840	0.58130	0.0650*	
H3B	0.48160	−0.16670	0.55770	0.0650*	
H5A	0.78820	−0.16100	0.76180	0.0520*	
H5B	0.76240	−0.34420	0.71300	0.0520*	
H6	0.91990	−0.04800	0.63970	0.0480*	
H7A	1.08780	−0.20780	0.73520	0.0640*	
H7B	1.14880	−0.22650	0.63670	0.0640*	
H7C	1.05130	−0.38190	0.68040	0.0640*	
H10A	0.61530	0.32520	0.73510	0.0460*	
H10B	0.48460	0.27250	0.80490	0.0460*	
H11	0.71090	0.45300	0.85770	0.0400*	
H13	0.43480	0.33540	0.97510	0.0540*	
H14	0.42200	0.24030	1.12820	0.0640*	
H15	0.64190	0.10180	1.19590	0.0690*	
H16	0.87520	0.04880	1.11640	0.0620*	
H19	1.11280	0.03880	0.99960	0.0610*	
H20	1.28690	0.04980	0.87390	0.0670*	
H21	1.20490	0.17560	0.74120	0.0630*	
H22	0.94320	0.30910	0.72900	0.0510*	

### Atomic displacement parameters (Å^2^)

**Table:**

	*U*^11^	*U*^22^	*U*^33^	*U*^12^	*U*^13^	*U*^23^
O8	0.0442 (8)	0.0714 (10)	0.0508 (7)	0.0151 (7)	−0.0161 (6)	−0.0121 (7)
O9	0.0444 (7)	0.0403 (7)	0.0363 (6)	0.0044 (6)	−0.0068 (5)	−0.0027 (5)
N1	0.0498 (9)	0.0414 (8)	0.0382 (8)	−0.0015 (7)	0.0020 (6)	−0.0056 (7)
N4	0.0417 (8)	0.0550 (9)	0.0435 (8)	0.0051 (8)	−0.0024 (6)	−0.0121 (8)
C2	0.0568 (12)	0.0583 (12)	0.0383 (8)	0.0044 (10)	−0.0124 (8)	−0.0084 (9)
C3	0.0476 (11)	0.0600 (13)	0.0542 (11)	0.0000 (9)	−0.0064 (9)	−0.0203 (10)
C5	0.0476 (10)	0.0462 (12)	0.0375 (9)	0.0095 (8)	0.0009 (7)	−0.0003 (7)
C6	0.0433 (9)	0.0379 (9)	0.0378 (8)	0.0011 (8)	−0.0045 (7)	−0.0016 (8)
C7	0.0459 (11)	0.0595 (12)	0.0541 (11)	0.0070 (10)	−0.0048 (9)	0.0006 (10)
C8	0.0344 (9)	0.0496 (11)	0.0355 (8)	−0.0001 (8)	0.0007 (7)	−0.0041 (8)
C10	0.0345 (9)	0.0386 (9)	0.0407 (9)	0.0062 (7)	−0.0036 (7)	−0.0001 (8)
C11	0.0320 (8)	0.0346 (10)	0.0340 (8)	0.0027 (7)	−0.0021 (6)	−0.0003 (6)
C12	0.0392 (9)	0.0299 (8)	0.0384 (8)	−0.0017 (7)	0.0004 (7)	−0.0035 (7)
C13	0.0439 (10)	0.0404 (10)	0.0497 (10)	−0.0027 (8)	0.0080 (8)	−0.0064 (8)
C14	0.0638 (13)	0.0478 (12)	0.0476 (11)	−0.0169 (10)	0.0196 (9)	−0.0120 (9)
C15	0.0811 (16)	0.0547 (13)	0.0357 (9)	−0.0228 (12)	0.0046 (10)	0.0017 (9)
C16	0.0641 (13)	0.0472 (12)	0.0425 (10)	−0.0114 (10)	−0.0101 (9)	0.0081 (8)
C17	0.0441 (10)	0.0325 (8)	0.0399 (9)	−0.0059 (7)	−0.0046 (7)	0.0014 (7)
C18	0.0365 (9)	0.0302 (8)	0.0440 (9)	0.0007 (7)	−0.0061 (7)	−0.0009 (7)
C19	0.0427 (11)	0.0424 (11)	0.0659 (12)	0.0049 (9)	−0.0119 (9)	0.0038 (9)
C20	0.0333 (10)	0.0463 (12)	0.0866 (16)	0.0063 (9)	−0.0055 (10)	−0.0070 (11)
C21	0.0379 (10)	0.0565 (12)	0.0641 (13)	−0.0028 (9)	0.0104 (9)	−0.0160 (10)
C22	0.0392 (9)	0.0469 (10)	0.0425 (9)	−0.0032 (8)	0.0013 (7)	−0.0044 (8)
C23	0.0323 (8)	0.0325 (8)	0.0397 (8)	0.0004 (7)	−0.0030 (6)	−0.0025 (7)
Cl1	0.0585 (3)	0.0495 (3)	0.0457 (2)	0.0006 (2)	0.0055 (2)	−0.0020 (2)

### Geometric parameters (Å, °)

**Table:**

O8—C8	1.208 (2)	C19—C20	1.384 (3)
O9—C8	1.353 (2)	C20—C21	1.384 (3)
O9—C10	1.436 (2)	C21—C22	1.395 (3)
N1—C2	1.495 (2)	C22—C23	1.381 (2)
N1—C6	1.495 (2)	C2—H2A	0.9600
N4—C3	1.464 (3)	C2—H2B	0.9600
N4—C5	1.457 (2)	C3—H3A	0.9600
N4—C8	1.366 (3)	C3—H3B	0.9600
N1—H1A	1.0600	C5—H5A	0.9600
N1—H1B	0.8800	C5—H5B	0.9600
C2—C3	1.508 (3)	C6—H6	0.9600
C5—C6	1.521 (2)	C7—H7A	0.9600
C6—C7	1.522 (3)	C7—H7B	0.9600
C10—C11	1.521 (2)	C7—H7C	0.9600
C11—C23	1.517 (2)	C10—H10A	0.9600
C11—C12	1.522 (2)	C10—H10B	0.9600
C12—C17	1.397 (2)	C11—H11	1.0900
C12—C13	1.386 (2)	C13—H13	0.9600
C13—C14	1.395 (3)	C14—H14	0.9600
C14—C15	1.384 (3)	C15—H15	0.9600
C15—C16	1.380 (3)	C16—H16	0.9600
C16—C17	1.390 (3)	C19—H19	0.9600
C17—C18	1.469 (2)	C20—H20	0.9600
C18—C23	1.394 (2)	C21—H21	0.9800
C18—C19	1.388 (2)	C22—H22	0.9600
			
Cl1···N1^i^	3.1353 (16)	H1B···H3A	2.5500
Cl1···C22	3.6222 (19)	H2A···H6	2.5500
Cl1···N1^ii^	3.1167 (16)	H2A···Cl1	3.1300
Cl1···H2A	3.1300	H2B···O8^vii^	2.4100
Cl1···H22	2.7200	H3A···H1B	2.5500
Cl1···H1B^i^	2.2600	H3A···H5B	2.5500
Cl1···H1A^ii^	2.0800	H3B···O8	2.3800
Cl1···H7B^ii^	2.9600	H3B···Cl1^vii^	3.0900
Cl1···H3B^iii^	3.0900	H5A···H14^x^	2.5300
O8···C2^iii^	3.316 (2)	H5A···H7A	2.5600
O9···C22	3.276 (2)	H5A···O9	2.2300
O8···H10B	2.6900	H5B···H7C	2.4800
O8···H2B^iii^	2.4100	H5B···H3A	2.5500
O8···H21^iv^	2.5400	H6···H2A	2.5500
O8···H10A	2.5700	H7A···H5A	2.5600
O8···H3B	2.3800	H7A···C21	3.0200
O9···H5A	2.2300	H7B···H1A	2.5000
N1···N4	2.829 (2)	H7B···Cl1^v^	2.9600
N1···Cl1^v^	3.1167 (16)	H7C···H1B	2.5000
N1···Cl1^vi^	3.1353 (16)	H7C···H5B	2.4800
N4···N1	2.829 (2)	H7C···H22^vi^	2.5900
C2···O8^vii^	3.316 (2)	H10A···O8	2.5700
C17···C19^viii^	3.470 (3)	H10B···O8	2.6900
C19···C17^ix^	3.470 (3)	H10B···C21^iv^	3.0500
C22···O9	3.276 (2)	H10B···H20^iv^	2.5700
C22···Cl1	3.6222 (19)	H10B···C15^xii^	3.1000
C5···H14^x^	2.9800	H10B···C13	3.0000
C10···H22	3.0800	H10B···C20^iv^	3.0200
C11···H19^viii^	3.0600	H11···C14^xii^	2.8700
C12···H19^viii^	2.9400	H11···H14^xii^	2.4300
C13···H10B	3.0000	H13···C16^xii^	2.8900
C14···H20^viii^	2.9800	H13···C15^xii^	2.8800
C14···H11^x^	2.8700	H14···H5A^xii^	2.5300
C15···H13^x^	2.8800	H14···H11^x^	2.4300
C15···H10B^x^	3.1000	H14···C5^xii^	2.9800
C16···H13^x^	2.8900	H16···C22^ix^	2.9600
C16···H19	3.0800	H19···C11^ix^	3.0600
C17···H19^viii^	2.9200	H19···C12^ix^	2.9400
C18···H19^viii^	3.0400	H19···C16	3.0800
C20···H10B^xi^	3.0200	H19···C18^ix^	3.0400
C21···H10B^xi^	3.0500	H19···C17^ix^	2.9200
C21···H7A	3.0200	H20···C14^ix^	2.9800
C22···H16^viii^	2.9600	H20···H10B^xi^	2.5700
H1A···Cl1^v^	2.0800	H21···O8^xi^	2.5400
H1A···H7B	2.5000	H22···H7C^i^	2.5900
H1B···H7C	2.5000	H22···Cl1	2.7200
H1B···Cl1^vi^	2.2600	H22···C10	3.0800
			
C8—O9—C10	114.92 (14)	C3—C2—H2B	110.00
C2—N1—C6	112.89 (14)	H2A—C2—H2B	109.00
C3—N4—C5	113.24 (16)	N4—C3—H3A	110.00
C3—N4—C8	117.98 (16)	N4—C3—H3B	109.00
C5—N4—C8	122.64 (17)	C2—C3—H3A	109.00
C2—N1—H1B	108.00	C2—C3—H3B	109.00
C6—N1—H1A	109.00	H3A—C3—H3B	109.00
C2—N1—H1A	107.00	N4—C5—H5A	109.00
H1A—N1—H1B	106.00	N4—C5—H5B	110.00
C6—N1—H1B	114.00	C6—C5—H5A	108.00
N1—C2—C3	109.43 (15)	C6—C5—H5B	109.00
N4—C3—C2	110.33 (15)	H5A—C5—H5B	109.00
N4—C5—C6	111.56 (15)	N1—C6—H6	109.00
C5—C6—C7	112.14 (15)	C5—C6—H6	109.00
N1—C6—C5	108.51 (14)	C7—C6—H6	108.00
N1—C6—C7	109.85 (16)	C6—C7—H7A	109.00
O9—C8—N4	110.82 (15)	C6—C7—H7B	111.00
O8—C8—O9	123.88 (19)	C6—C7—H7C	109.00
O8—C8—N4	125.26 (17)	H7A—C7—H7B	110.00
O9—C10—C11	107.66 (13)	H7A—C7—H7C	109.00
C10—C11—C12	117.54 (13)	H7B—C7—H7C	109.00
C12—C11—C23	101.91 (12)	O9—C10—H10A	110.00
C10—C11—C23	114.66 (13)	O9—C10—H10B	109.00
C11—C12—C13	128.75 (15)	C11—C10—H10A	111.00
C13—C12—C17	120.70 (16)	C11—C10—H10B	110.00
C11—C12—C17	110.36 (14)	H10A—C10—H10B	110.00
C12—C13—C14	118.12 (18)	C10—C11—H11	103.00
C13—C14—C15	121.0 (2)	C12—C11—H11	113.00
C14—C15—C16	121.04 (19)	C23—C11—H11	107.00
C15—C16—C17	118.5 (2)	C12—C13—H13	119.00
C12—C17—C16	120.66 (17)	C14—C13—H13	123.00
C16—C17—C18	130.86 (17)	C13—C14—H14	120.00
C12—C17—C18	108.42 (14)	C15—C14—H14	119.00
C17—C18—C23	108.85 (14)	C14—C15—H15	120.00
C19—C18—C23	120.38 (16)	C16—C15—H15	119.00
C17—C18—C19	130.77 (16)	C15—C16—H16	120.00
C18—C19—C20	118.94 (19)	C17—C16—H16	121.00
C19—C20—C21	120.64 (17)	C18—C19—H19	119.00
C20—C21—C22	120.70 (18)	C20—C19—H19	122.00
C21—C22—C23	118.58 (17)	C19—C20—H20	121.00
C11—C23—C22	128.88 (15)	C21—C20—H20	119.00
C18—C23—C22	120.72 (15)	C20—C21—H21	117.00
C11—C23—C18	110.40 (14)	C22—C21—H21	122.00
N1—C2—H2A	109.00	C21—C22—H22	121.00
N1—C2—H2B	110.00	C23—C22—H22	120.00
C3—C2—H2A	109.00		
			
C10—O9—C8—O8	−17.4 (2)	C11—C12—C13—C14	−173.75 (18)
C10—O9—C8—N4	164.52 (14)	C17—C12—C13—C14	0.6 (3)
C8—O9—C10—C11	−167.41 (13)	C11—C12—C17—C16	175.50 (16)
C6—N1—C2—C3	−57.2 (2)	C11—C12—C17—C18	−2.04 (17)
C2—N1—C6—C5	55.8 (2)	C13—C12—C17—C16	0.2 (3)
C2—N1—C6—C7	178.70 (16)	C13—C12—C17—C18	−177.31 (16)
C5—N4—C3—C2	−57.0 (2)	C12—C13—C14—C15	−0.7 (3)
C8—N4—C3—C2	96.2 (2)	C13—C14—C15—C16	0.1 (3)
C3—N4—C5—C6	56.7 (2)	C14—C15—C16—C17	0.7 (3)
C8—N4—C5—C6	−95.1 (2)	C15—C16—C17—C12	−0.8 (3)
C3—N4—C8—O8	12.5 (3)	C15—C16—C17—C18	176.07 (18)
C3—N4—C8—O9	−169.49 (15)	C12—C17—C18—C19	179.93 (18)
C5—N4—C8—O8	163.05 (18)	C12—C17—C18—C23	0.59 (18)
C5—N4—C8—O9	−19.0 (2)	C16—C17—C18—C19	2.7 (3)
N1—C2—C3—N4	55.6 (2)	C16—C17—C18—C23	−176.61 (18)
N4—C5—C6—N1	−54.2 (2)	C17—C18—C19—C20	178.81 (18)
N4—C5—C6—C7	−175.72 (18)	C23—C18—C19—C20	−1.9 (3)
O9—C10—C11—C12	−74.83 (17)	C17—C18—C23—C11	1.10 (18)
O9—C10—C11—C23	44.87 (17)	C17—C18—C23—C22	−178.39 (16)
C10—C11—C12—C13	−56.5 (2)	C19—C18—C23—C11	−178.32 (16)
C10—C11—C12—C17	128.77 (15)	C19—C18—C23—C22	2.2 (3)
C23—C11—C12—C13	177.33 (17)	C18—C19—C20—C21	0.2 (3)
C23—C11—C12—C17	2.55 (16)	C19—C20—C21—C22	1.3 (3)
C10—C11—C23—C18	−130.26 (14)	C20—C21—C22—C23	−1.0 (3)
C10—C11—C23—C22	49.2 (2)	C21—C22—C23—C11	179.91 (18)
C12—C11—C23—C18	−2.18 (16)	C21—C22—C23—C18	−0.7 (3)
C12—C11—C23—C22	177.26 (17)		

Symmetry codes: (i) *x*, *y*+1, *z*; (ii) −*x*+2, *y*+1/2, −*z*+1; (iii) −*x*+1, *y*+1/2, −*z*+1; (iv) *x*−1, *y*, *z*; (v) −*x*+2, *y*−1/2, −*z*+1; (vi) *x*, *y*−1, *z*; (vii) −*x*+1, *y*−1/2, −*z*+1; (viii) −*x*+2, *y*+1/2, −*z*+2; (ix) −*x*+2, *y*−1/2, −*z*+2; (x) −*x*+1, *y*−1/2, −*z*+2; (xi) *x*+1, *y*, *z*; (xii) −*x*+1, *y*+1/2, −*z*+2.

### Hydrogen-bond geometry (Å, °)

**Table:**

*D*—H···*A*	*D*—H	H···*A*	*D*···*A*	*D*—H···*A*
N1—H1A···Cl1^v^	1.06	2.08	3.117 (2)	165.
N1—H1B···Cl1^vi^	0.88	2.26	3.135 (2)	171.
C2—H2B···O8^vii^	0.96	2.41	3.316 (2)	157.
C3—H3B···O8	0.96	2.38	2.778 (3)	104.
C5—H5A···O9	0.96	2.23	2.665 (2)	106.
C21—H21···O8^xi^	0.98	2.54	3.469 (2)	158.
C22—H22···Cl1	0.96	2.72	3.622 (2)	157.

Symmetry codes: (v) −*x*+2, *y*−1/2, −*z*+1; (vi) *x*, *y*−1, *z*; (vii) −*x*+1, *y*−1/2, −*z*+1; (xi) *x*+1, *y*, *z*.

## References

[bb1] ACD (2011). *ACD/Labs* Advanced Chemistry Development Inc., Toronto, Ontario, Canada.

[bb2] Altomare, A., Burla, M. C., Camalli, M., Cascarano, G. L., Giacovazzo, C., Guagliardi, A., Moliterni, A. G. G., Polidori, G. & Spagna, R. (1999). *J. Appl. Cryst.* **32**, 115–119.

[bb3] Cho, Y. S., Borland, M., Brain, C., Chen, C. H.-T., Cheng, H., Chopra, R., Chung, K., Groarke, J., He, G., Hou, Y., Kim, S., Kovats, S., Lu, Y., OReilly, M., Shen, J., Smith, T., Trakshel, G., Vogtle, M., Xu, M., Xu, M. & Sung, M. J. (2010). *J. Med. Chem.* **53**, 7938–57. 10.1021/jm100571n 21038853

[bb4] Flack, H. D. (1983). *Acta Cryst.* A**39**, 876–881.

[bb5] Johnson, C. K. (1976). *ORTEPII* Oak Ridge National Laboratory, Tennessee, USA

[bb6] Kitaigorodskij, A. I. (1973). In *Molecular Crystals and Molecules* New York: Academic Press.

[bb7] MacKay, S., Edwards, C., Henderson, A., Gilmore, C., Stewart, N., Shankland, K. & Donald, A. (2000). The *maXus* program package. Chemistry Department, The University, Glasgow, Scotland, MacScience Co., Japan, and Nonius BV, The Netherlands.

[bb8] Nonius (1998). *COLLECT* Nonius BV, Delft, The Netherlands.

[bb9] Otwinowski, Z. & Minor, W. (1997). *Methods in Enzymology*, Vol. 276, *Macromolecular Crystallography*, Part A, edited by C. W. Carter, Jr & R. M. Sweet, pp. 307–326, New York: Academic Press.

[bb10] Sheldrick, G. M. (2008). *Acta Cryst.* A**64**, 112–122. 10.1107/S0108767307043930 18156677

[bb11] Spek, A. L. (2009). *Acta Cryst.* D**65**, 148–155. 10.1107/S090744490804362X PMC2631630 19171970

[bb12] Wang, T., Yin, Z., Zhang, Z., Bender, J. A., Yang, Z., Johnson, G., Yang, Z., Zadjura, L. M., Arienzo, C. J., DiGiugno Parker, D., Gesenberg, C., Yamanaka, G. A., Gong, Y.-F., Ho, H.-T., Fang, H., Zhou, N., McAuliffe, B. V., Eggers, B. J., Fan, L., Nowicka-Sans, B., Dicker, I. B., Gao, Q., Colonno, R. J., Lin, P.-F., Meanwell, N. A. & Kadow, J. F. (2009). *J. Med. Chem.* **52**, 7778–7787. 10.1021/jm900843g 19769332

